# Tumor Copy Number Alteration Burden as a Predictor for Resistance to Immune Checkpoint Blockade across Different Cancer Types

**DOI:** 10.3390/cancers16040732

**Published:** 2024-02-09

**Authors:** Karama Asleh, Rodney J. Ouellette

**Affiliations:** 1 Department of Pathology and Laboratory Medicine, Halifax, NS B3H 1V8, Canada; 2 Beatrice Hunter Cancer Research Institute, Halifax, NS B3H 0A2, Canada; rodneyo@canceratl.ca; 3 Atlantic Cancer Research Institute, Moncton, NB E1C 8X3, Canada; 4 Department of Chemistry and Biochemistry, Université de Moncton, Moncton, NB E1A 3E9, Canada; 5 Dr. Georges L. Dumont University Hospital, Vitalité Health Network, Moncton, NB E1C 2Z3, Canada

**Keywords:** copy number alteration burden, tumor mutational burden, immune checkpoint blockade, next-generation sequencing, MSK-IMPACT assay, predictive biomarkers

## Abstract

**Simple Summary:**

Drugs that help the immune system attack cancer, known as immunotherapy, benefit only a small fraction of patients with cancer that has spread distant from the primary organ. Thus, markers that indicate immunotherapy failure are needed. Overall changes to DNA structure that lead to the gain or loss of extra copies of genes are known as copy number alteration (CNA) burden. In this study, we obtained data on CNA burden, clinical information, and overall tumor mutations, known as tumor mutational burden (TMB), for 1661 patients. These patients were all treated with immunotherapy and had their cancer specimens analyzed by an approved clinical test called MSK-IMPACT. We report that CNA burden could predict those patients who do not benefit from immunotherapy. Additionally, tumors with high CNA but low TMB could identify those with the worst survival. This work can better match patients to their most effective treatment of immunotherapy or other therapies.

**Abstract:**

Immune checkpoint blockade (ICB) benefits only a subset of advanced cancer patients, and predictive biomarkers for immunotherapy response are needed. Recently, copy number alteration (CNA) burden has been proposed to predict ICB resistance. We assessed this finding using the publicly accessible data for 1661 ICB-treated patients whose tumors were profiled by MSK-IMPACT, an approved targeted assay in clinical care. We tested the hypothesis that the continuous increase in CNA burden is associated with poor overall survival following ICB. In addition, we hypothesized that the combinatorial biomarkers of tumor mutational burden (TMB) and CNA burden would better stratify patients for immune status and ICB response. Of the 1661 cases, 79% (*n* = 1307) were treated with anti PD-1/PD-L1 and the remaining 21% (*n* = 354) with anti CTLA-4 or the combination of both. In a multivariate analysis, increase in CNA burden was associated with poor overall survival [HR = 1.52, 95% CI (1.01–2.30), *p* = 0.04]. The combination of biomarkers TMB and CNA burden stratified patients into four clinically distinct subsets among which “LowTMB/HighCNA” showed the worst survival (*p* < 0.0001). The four patient subsets had unique CNA profiles and enriched pathways, which could predict transcriptional and phenotypic effects related to immune signaling and CD8+ T-cell abundance in the tumor microenvironment. CNA burden was associated with poor overall survival in patients receiving ICB and could improve patient stratification when incorporated with TMB. These findings may guide patient selection for immunotherapy or alternative strategies.

## 1. Introduction

With the advent of next-generation sequencing (NGS) assays, the list of targeted therapies that act on specific molecular alterations has exponentially grown over the past decade [1,2]. The shift from a site-specific to genetic-specific approach is transforming the field of precision oncology, with several tissue-agnostic biomarkers being proposed in advanced cancers [3,4,5]. Indeed, many of these tissue-agnostic biomarkers have obtained their regulatory approvals based on a proven efficacy in clinical trials across different cancer histologies [4]. In 2020, tumor mutational burden (TMB) received an accelerated US Food and Drug Administration (FDA) approval as a tissue-agnostic biomarker for immunotherapy based on a significant benefit observed in a prospective–retrospective analysis of the KEYNOTE-158 trial across multiple advanced solid tumors [6,7]. These findings, along with data showing that TMB better associates with immunotherapy response when compared to simple PD-L1 immunohistochemical (IHC) assessment [8,9,10], have driven the paradigm shift toward endorsing TMB in the current treatment guidelines.

Immunotherapy is continuously expanding the arsenal of genomically driven therapeutic options through the incorporation of TMB as an important decision-making tool in clinical practice [10,11]. Targeted NGS panels have been recently developed [9] to evaluate TMB more easily in clinical practice when compared to the more expensive and complex whole-exome sequencing assays. Currently, the two NGS FDA-approved companion diagnostics for immunotherapy agents of FoundationOne and the MSKCC (MSK-IMPACT) can be used on tumor tissue biopsy to determine TMB in routine clinical care [12,13,14,15]. The MSK-IMPACT assay identifies somatic exonic mutations and copy number alterations (CNAs) in a predefined set of 468 cancer-related genes and has a proven capacity for predicting immunotherapy response [16,17]. In a retrospective analysis that included 1662 patients with advanced solid tumors of multiple origins profiled by the MSK-IMPACT assay, a significant association between higher TMB and improved overall survival (OS) on immune checkpoint blockade (ICB) was reported [17].

To date, several cut-points have been proposed for TMB estimation [9], with the recent consensus being 10 mut/mb or greater for universally defining high TMB tumors with response to immunotherapy regardless of cancer type [6]. While TMB has been originally developed to expand patients’ selection for immunotherapy beyond the sole assessment of IHC PD-L1, survival benefits are still only seen in a limited subset of cancer patients [18,19,20]. Achieving a significantly durable clinical response on ICB is a pressing challenge not only due to analytical variability issues that still exist across TMB and PD-L1 assessment platforms [9] but mostly resulting from a complex network of biological pathways eliciting innate and acquired therapeutic resistance in the patient’s tumor microenvironment (TME) [18,21,22]. Thus, it is not surprising that recent efforts have outlined a framework that guides future immune-resistance research through defining eight biological processes, referred to as immune resistance nodes, to identify biomarkers that explain the high rates of failures still observed with immunotherapy [23]. The application of this framework specifically calls for the integration of omics-based analyses beyond TMB to provide insights into the selection of biomarkers that can be exploited for tailoring better therapeutic strategies in the clinic [23].

At the genomic level, multiple analyses of the copy number alteration (CNA) repertoire in advanced cancers have contributed to the understanding of mechanisms underlying patient’s resistance to immunotherapy, with several CNA patterns being reported that could be linked to ICB response [24,25,26,27]. More specifically, a CNA burden that reflects the fraction of gains and losses across the profiled genome has been recently reported to be a predictor for poor response to ICB in several tumors including melanoma, non-small-cell lung cancer (NSCLC), and gastrointestinal cancer [25,28,29,30]. However, these studies mainly investigated CNA burden using whole-exome sequencing methods not widely utilized in routine clinical care. Furthermore, high CNA burden determined by these assays is characterized by numerous low-frequency altered genes, which creates challenges for the development of clinically useful biomarkers. As such, the determination of CNA burden from currently approved genomic-based tests would provide more readily available information to guide therapeutic choices in the clinic.

In this study, we evaluated CNA burden as a predictor for response to immunotherapy. As our data source, we analyzed a large, publicly available series containing ICB treatment outcome information for 1661 patients with advanced cancers profiled by MSK-IMPACT [16,17]. We tested the hypothesis that high CNA burden, as measured by the MSK-IMPACT assay, is associated with poor overall survival after treatment with ICB. In addition, we hypothesized that the combinatorial biomarkers of TMB and CNA burden can stratify patients into distinct subsets with differential response to ICB. We further investigated individual CNAs characteristic of these subsets, correlating results in the context of biological processes and immune resistance mechanisms to inform personalized treatment-biomarker-driven clinical trial designs.

## 2. Material and Methods

### 2.1. Study Population and Design

**Figure 1:**
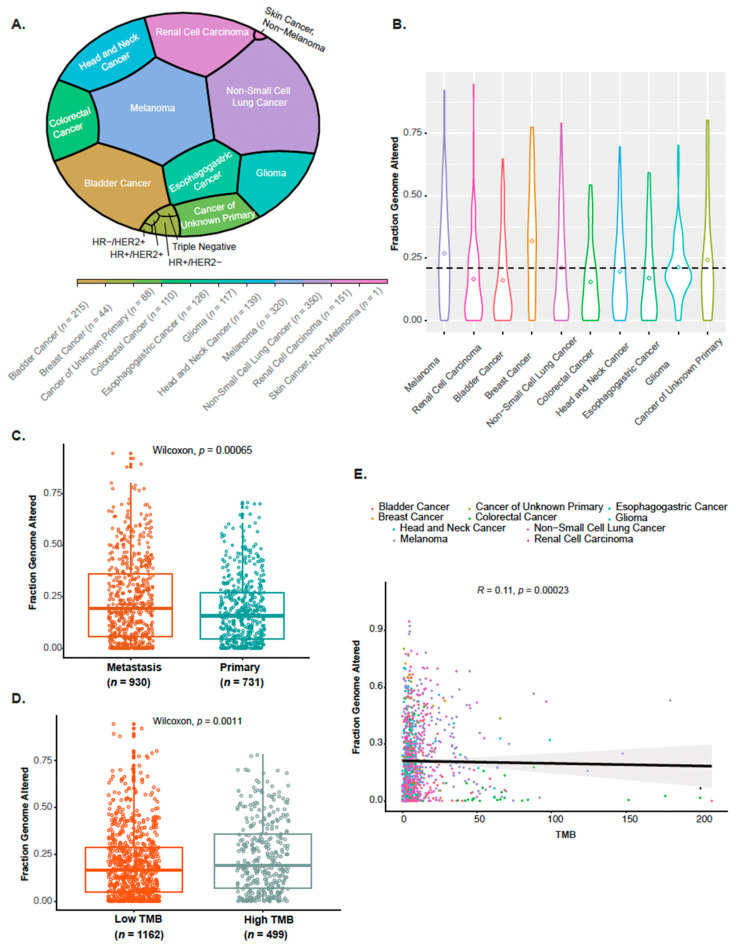
Characteristics of the translational study cohort of the MSK-IMPACT treated with immune checkpoint blockade. (**A**) Vornoi treemap showing the distribution of the total of 1661 cases across different tumor types. (**B**) CNA burden, as determined by the fraction of genome altered values, across the different tumor types. Violin plots show the median (center bar), and the horizontal dotted line displays the base mean of all. (**C**) Fraction of genome altered distribution according to specimen type analyzed. Boxplots show the median (center bar), the third (upper edge), and first quartiles (lower edge) of FGA scores. Each point represents one case. Pair-wise comparisons were performed with the two-sided Wilcoxon rank-sum test. (**D**) Fraction of genome altered distribution according to tumor mutational burden (TMB). Box plots show the median (center bar), the third (upper edge), and first quartiles (lower edge) of the FGA scores. The universal prespecified FDA-approved cut-point of 10 mut/mb or greater was used to define “HighTMB” vs. “LowTMB” tumors. Each point represents one case. Pair-wise comparisons were performed with the two-sided Wilcoxon rank-sum test. (**E**) Scatter plot showing the correlation between the continuous expression of TMB and CNA burden as determined by fraction of genome altered scores. Results were derived from the 2-sided Pearson correlation coefficient (r) test.

Data from 1661 cancer patients previously subjected to the FDA-authorized MSK-IMPACT profiling (mostly as part of the clinical trial NCT01775072, with the remainder as part of routine clinical care) and who had received at least one dose of ICB therapy were obtained from Samstein et al. in Nat. Genet. 2019 [17] and constituted the translational cohort of this study. Most of these cases (representing 94% of tumors excluding glioma; *n* = 1446) had stage IV or metastatic disease, while a small number of patients had a locoregional recurrent disease (*n* = 10) or stage III melanoma (*n* = 98). Of the total 1661 patients included, 1307 (79%) were treated with anti-PD-1 or -PD-L1 therapy, 99 (6%) received anti-CTLA-4, and 255 (15%) were treated with the combination of anti-CTLA-4 and anti-PD-1/PD-L1 therapies. These 1661 cases spanned 10 different major tumor types, comprising 350 NSCLC cases (21%), 320 melanoma cases (19%), 215 bladder cancers (13%), 139 head and neck cancers (8%), and others (Table 1, Figure 1A).

### 2.2. Statistical Analysis of the MSK-IMPACT Translational Cohort

To test the primary hypothesis on whether increased FGA expression is associated with poor overall survival after ICB treatment, univariate and multivariate survival analyses were performed using Cox regression models and stratified log-rank tests. The associations between hazard ratio (HR) and 95% confidence interval (CI) were calculated, and the results were displayed using forest plots. Variables included in the multivariate model were age (continuous), sex (male vs. female), sample type (primary vs. metastasis), and treatment type (anti-PD-1/PD-L1 vs. anti-CTLA-4 vs. combination). When relevant, results were further adjusted to smoking history for NSCLC and receptor status (according to hormonal receptor and HER2) for breast cancer. Analyses evaluating the secondary hypothesis of the combined expression of TMB and CNA burden used Kaplan–Meier curves to display survival outcomes according to biomarker expression status. The universal prespecified and established FDA-approved cut-point of 10 mut/mb was used to define “HighTMB” vs. “LowTMB” tumors. For CNA burden, we defined subgroups by the median cut-point within each tumor type since CNA burden varied across different tumor histologies. Exploratory analyses evaluating associations between individual copy number alterations within each distinct "TMB/CNA" subgroup vs. others were tested using Fisher’s test, with balloon plots used to display results. The *χ*^2^ test was used to assess associations between categorical variables. The assessment of continuous variables against categorical variables was performed using Wilcoxon rank-sum tests for pair-wise comparisons and Kruskal–Wallis for three categories and above. The correlation between continuous variables was tested using the Pearson correlation coefficient (r). All tests were performed 2-sided at a significance level of 0.05 using R statistical software (version #4.1.2).

### 2.3. Assessment of Tumor Mutational and Copy Number Alteration Burden

TMB was defined as the number of nonsynonymous mutations divided by the number of megabases (mbs) in the coding region captured by the MSK-IMPACT assay as previously described [17,31]. Data on CNA burden for the translational study cohort were obtained through the cBioPortal for Cancer Genomics database [32]. CNA burden was defined as the fraction of genome altered (FGA) and calculated as the log2 copy number variation (gain or loss) >0.2 divided by the size of genome whose copy number was profiled [31]. Data on individual copy number alterations, identified in an earlier version of the MSK-IMPACT assay based on a 410-gene panel, were retrieved from Zehir et al. Nat. Med. [16]. The levels of copy number per gene were defined according to a previous publication in the following fashion: −2, deep deletion; −1, shallow deletion; 0, diploid; 1, low-level gain; and 2, high-level amplification [32].

### 2.4. Analysis of CNA Association with Immune Cell Abundance and Cofunctionality Network

The association between individual CNAs and the immune cell abundance of CD8+ T cells was evaluated in The Cancer Genome Atlas (TCGA) cohorts using the TIMER2.0 tool, a comprehensive resource of deconvolution method for the estimation of immune infiltrate populations [33]. The log-fold changes of immune CD8+ T-cell infiltration levels between the specified alteration group (gain/amplification or deletion) and the normal one (diploid) were compared using Wilcoxon rank-sum tests. Results with log2FC > 0 indicated a higher level of CD8+ T-cell infiltrates, while log2FC < 0 indicated a lower level of CD8+ T-cell infiltrates in the alteration group.

Prediction of gene functionality and biological processes based on CNAs were performed using a guilt-by-association strategy as part of a comprehensive network created using 23,372 well-described functional gene sets reported by Bhattacharya et al. in Nat. Commun. [34]. Z scores were used for gene expression values, and the cofunctionality correlation between genes was displayed using a scale from 0 to 1. Enriched biological processes for the predicted altered gene expression were derived from Reactome and their predicted phenotypic effects from the Mammalian Phenotype Ontology database available at http://195.240.45.156//GuiltByAssociation/ (accessed on 27 November 2023) [34].

### 2.5. Data Availability

This study involved the collection and analysis of data from multiple publicly available datasets. The MSK-IMPACT cancer data analyzed can be accessed through the cBioPortal for Cancer Genomics repository (https://www.cbioportal.org/; accessed on 15 June 2023—unique identifier: “TMB and Immunotherapy (MSK, Nature Genetics 2019)”); Supplementary Data were obtained from Samstein et al. in Nat. Genet. 2019 [17] and Zehir et al. Nat. Med. [16]. The association between individual CNAs and the immune cell abundance of CD8+ T cells in TCGA cohorts was evaluated using the TIMER2.0 tool [33], which is available online (http://timer.comp-genomics.org/timer/) and was accessed on 3 December 2023. The cofunctionality network analysis and phenotypic effects prediction of individual genes based on CNAs were performed using the framework [34] available at http://195.240.45.156//GuiltByAssociation/, which was accessed on 27 November 2023. Data investigating the incorporation of CNAs in biomarker-driven clinical trial designs were retrieved from the AACR Project GENIE Consortium database [2] through the website https://www.mycancergenome.org, which was accessed on 13 October 2023.

## 3. Results

Of the 1661 patients included in the translational study, 930 (56%) had their archival tumor tissue specimens obtained from metastatic sites and profiled through the MSK-IMPACT assay (Table 1). Using the universal cut-point of 10 mut/mb or greater to define “HighTMB” vs. “LowTMB” tumors, 499 (30%) of the patients were assigned as having “HighTMB” tumors (Table 1). CNA burden, as determined by FGA scores, varied across the different tumor types, and displayed a mean of FGA = 0.21 for all cancers (Figure 1B).

### 3.1. Association of CNA Burden with Metastasis and TMB

When examining the CNA burden according to specimen type, FGA scores were significantly higher in tissues obtained from metastatic sites when compared to primary (Wilcoxon *p* < 0.001) (Figure 1C). Interestingly, FGA values of 0.75 and above were only observed in metastatic specimens (Figure 1C) illustrating the higher complexity of CNAs in metastatic sites [35]. Higher TMB scores were further observed to be significantly associated with metastatic specimens (Wilcoxon *p* < 0.001).

When assessing the correlation between CNA burden and TMB, FGA scores were significantly higher in tumors classified as “HighTMB” (Wilcoxon *p* = 0.001). However, the correlation between FGA scores and TMB as continuous variables denoted a significantly low correlation (Pearson r = 0.11, *p* < 0.001) (Figure 1D,E).

### 3.2. Correlation of CNA Burden with Overall Survival after Immune Checkpoint Blockade in the Whole Cohort and across Tumor Types

**Figure 2:**
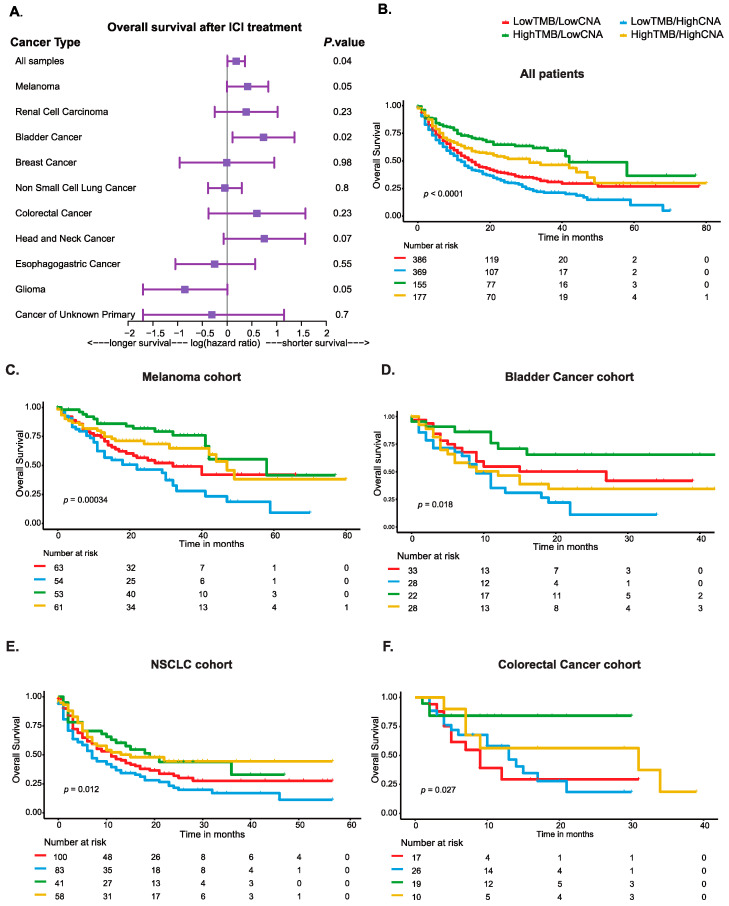
Survival analyses showing the overall survival for patients included in the translational study cohort of the MSK-IMPACT treated with immune checkpoint blockade. (**A**) Forest plot for the continuous expression values of CNA burden, as determined by fraction of genome altered scores, across all cases and within each cancer type included. Hazard ratios, 95% confidence intervals, were *p*-values are derived from Cox regression multivariate analysis adjusted for age, sex, sample type, and treatment type, and when relevant, results were further adjusted for smoking history for NSCLC and for receptor status for breast cancer. (**B**) Kaplan–Meier curves showing overall survival for cancer patients stratified according to the combinatorial expression of TMB and CNA burden categories in all cases and in (**C**) the melanoma cohort, (**D**) bladder cancer cohort, (**E**) NSCLC cohort, and (**F**) colorectal cancer cohort.

In a multivariate analysis testing the primary hypothesis of CNA burden as a continuous variable, an increase in the scores of FGA was associated with a significantly lower OS after treatment with ICB (HR, 1.52; 95% CI, 1.01–2.30; *p* = 0.04). This association was further observed in melanoma and bladder cancer with a trend toward a lower OS in head and neck cancers (Figure 2A; Supplementary Table S1). Interestingly, the increase in FGA scores among glioma patients was found to be associated with a favorable OS after ICB (Figure 2A; Supplementary Table S1).

### 3.3. Combined Biomarkers of TMB and CNA Burden Stratified Patients with Distinct Survival Outcomes after Immune Checkpoint Blockade Treatment

Testing our secondary hypothesis that the combination of TMB and CNA burden can better stratify patients into subsets with differential response to ICB revealed that tumors with low TMB but high CNA burden, “LowTMB/HighCNA”, showed the worst OS when compared to other subgroups (HR, 1.6; 95% CI, 1.37–1.88; *p* < 0.0001) (Figure 2B). In contrast, the subgroup with high TMB but low CNA burden, “HighTMB/LowCNA”, exhibited the best OS (log-rank *p* < 0.0001) (Figure 2B). The subgroups with high or low scores for both TMB and CNA burden (i.e., “LowTMB/LowCNA” and “HighTMB/HighCNA”) were characterized with intermediate OS after immunotherapy (Figure 2B). These observations were also found to be significant in melanoma, bladder cancer, NSCLC, and colorectal cancer (Figure 2C,D). Among these tumors, when CNA burden was evaluated as an individual biomarker (i.e., “LowCNA” vs. “HighCNA”), the results were significant only in the whole cohort, melanoma, and bladder cancer (Supplementary Figure S1A–E).

### 3.4. Individual Key Copy Number Alterations Characterized Four Tumor Subsets with Differential Survival Outcomes after Immune Checkpoint Blockade Treatment

We next aimed to characterize the four subgroups we identified in terms of differential survival outcomes after ICB. We first examined the distributions of different tumor types across these subgroups and found that melanoma, NSCLC, and bladder cancers constituted most of the cases in the subgroups that had high TMB. In contrast, less immunogenic tumors of renal cell carcinoma (RCC) and head and neck, glioma, esophagogastric, and breast cancer were mainly included in the subgroups that had low TMB. Colorectal cancers had a mix of both high TMB and low TMB cases and were distributed across the four “TMB/CNA” subgroups (Supplementary Figure S2; Supplementary Table S2). In addition, a higher fraction of tumor tissue specimens obtained from metastatic sites were observed in the subgroups that had high TMB (Supplementary Table S3). Additional characteristics of these four subgroups are displayed in Supplementary Table S3.

**Figure 3:**
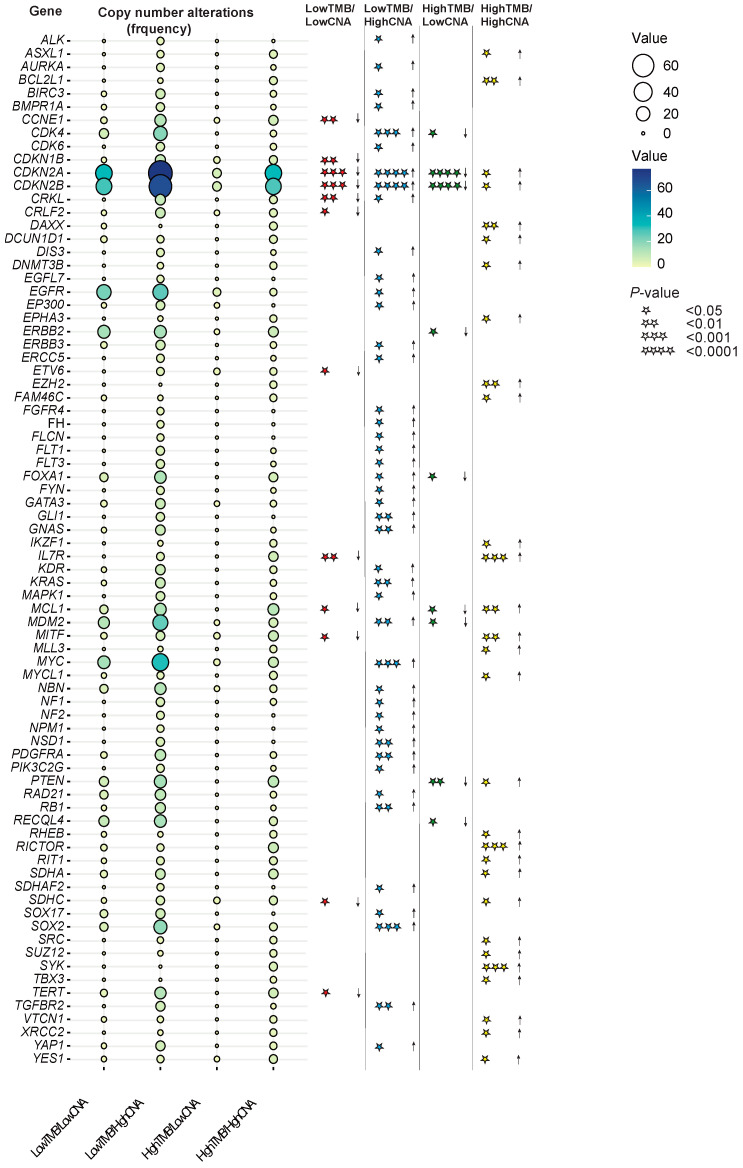
Balloon plot depicting the 78 genes whose alterations were characteristic of the four “TMB/CNA” subgroups with their frequencies and significant associations for each subgroup vs. those of others. The list of 78 genes was derived from testing the CNAs of each of the individual 410 genes in each subgroup vs. those of others using Fisher’s test. Significance levels are displayed with asterisks as follows: * *p* < 0.05, ** *p* < 0.01, *** *p* < 0.001, and **** *p* < 0.0001.

To examine whether individual CNAs would be characteristic for at least one of the subgroups identified, we compared the alterations frequency for the single 410 genes included in the MSK-IMPACT assay within each “TMB/CNA” subgroup vs. those of others. Among these 410 genes, individual alterations in 78 were found to significantly distinguish at least one subgroup vs. others (Fisher’s test *p* ≤ 0.05) (Supplementary Table S4). A summary of these 78 genes with their frequencies and significant associations for each subgroup vs. others are depicted in Figure 3. In this analysis, the “LowTMB/HighCNA” subgroup which displayed the worst survival was characterized with significantly higher amplifications and losses in genes related to several pathways including cell cycle, receptor tyrosine kinase, DNA repair, TGFβ signaling, MAPK signaling, MYC pathway, and others (Supplementary Table S5). In contrast, the subgroup “HighTMB/LowCNA”, which exhibited the best survival, showed a copy number profile associated with significantly lower alterations in these genes (Supplementary Table S5). While the subgroup “HighTMB/HighCNA” showed some pathways in common with “LowTMB/HighCNA”, the subgroup was more characterized with higher alterations in genes related to chromatin remodeling and DNA methylation, tricarboxylic acid cycle and metabolic reprogramming, PI3K/AKT1/mTOR pathway, immune response, and the T-cell coinhibitory molecule B7-H4 (Supplementary Table S5). However, the subgroup “LowTMB/LowCNA” demonstrated a significantly lower degree of alteration in genes involved in these pathways (Supplementary Table S5).

### 3.5. Distribution of Key Copy Number Alterations across Different Tumor Types and Their Association with Reduced CD8+ T-Cell Infiltration

In the analysis of the distribution of key copy number alterations in the 78 genes found to be characteristic of the four “TMB/CNA” subgroups, the main findings revealed that *EGFR* amplification was the most frequent alteration and was more prevalent in bladder cancer, followed by RCC. The receptor tyrosine kinase *ERBB2* was primarily amplified in bladder cancer, while *FGFR4* and *EGFL7* alterations were mainly seen among RCC cases. *MYC* amplification and *PTEN* loss were mostly prevalent in breast tumors, while amplifications for *SOX2* were mostly observed in melanoma (Supplementary Figure S3).

Since high CNA burden was associated with poor survival outcomes in our analysis, we next examined whether individual alterations in the 78 genes that were found to be characteristic of the four “TMB/CNA” subgroups may be correlated with the decreased cytotoxic CD8+ T-cell infiltration that is known to be linked with lower survival rates in immunotherapy [36]. We restricted the analysis to the same cancer types in TCGA that represented the composition of tumor cases in our dataset. Interestingly, majority of the alterations in the 78 genes displayed a significantly negative correlation with CD8+ T-cell infiltration in at least one tumor type (Supplementary Table S6). Higher numbers of alterations that were found to be associated negatively with CD8+ T cells were observed most commonly in breast cancer (26/78), followed by esophagogastric cancer (25/78) (Supplementary Figure S4, Supplementary Table S6). Interestingly *MYC* and *RB1* were the most frequently altered genes that were associated significantly with a lower abundance of CD8+ T-cell infiltrates across different tumor types (Supplementary Table S6).

**Figure 4:**
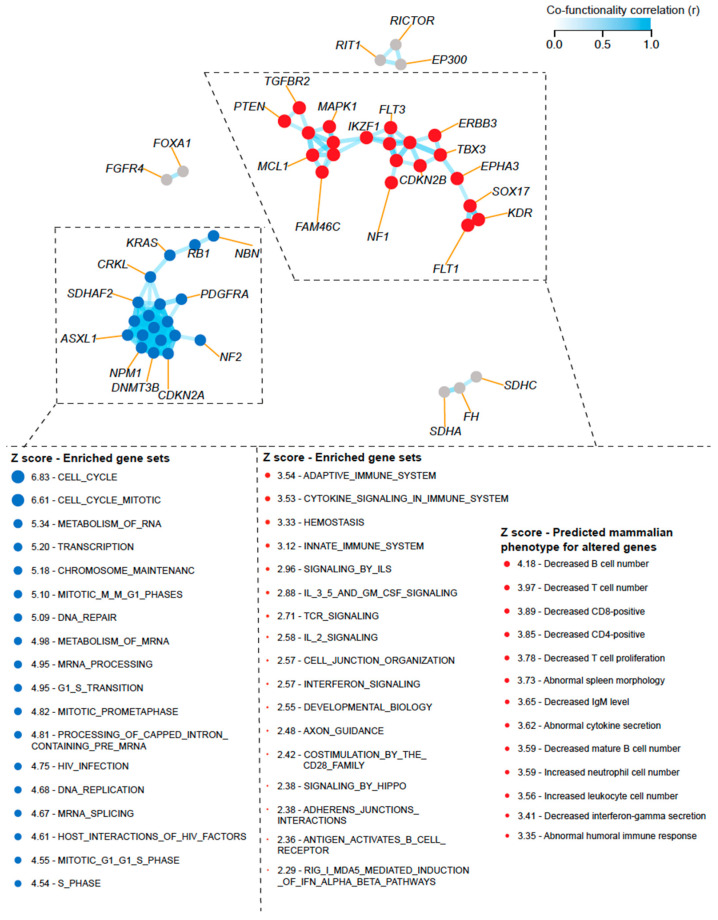
Constructed cofunctionality network for the individual alterations that were found to be characteristic of the subgroups with high CNA burden (“LowTMB/HighCNA” and “HighTMB/HighCNA”). Results were generated using an integrative tool that predicts gene functions based on CNA profiles following a guilt-by-association strategy as previously published by Bhattacharya et al. in Nat. Commun. [34]. Two main clusters are shown with one enriched for genes predicted to be involved in biological processes related to cell cycle and DNA replication and repair, whereas another cluster was enriched for genes associated with immunological processes (e.g., adaptive immune response, cytokine and interferon signaling, and T-cell receptor signaling). The altered expression levels of genes in the “immune cluster” were predicted to result in decreased abundance of T cells, CD8+ T cells, and interferon gamma secretion. Enriched biological processes for the predicted altered gene expression were derived from Reactome and their predicted phenotypic effects from Mammalian Phenotype Ontology database.

Given the lack of paired RNA expression data available for the corresponding altered genes in the MSK-IMPACT dataset, we sought to investigate how CNAs can transcriptionally activate biological processes and affect the activity and composition of immune cells present in the TME. We constructed a cofunctionality network for the individual alterations that were found to be characteristic of the subgroups with high CNA burden (i.e., “LowTMB/HighCNA” and “HighTMB/HighCNA”) using an integrative tool that predicts gene functions based on CNA profiles following a previously published guilt-by-association strategy [34]. We identified two main clusters with one enriched for genes predicted to be involved in the biological processes related to cell cycle and DNA replication and repair, whereas another cluster was enriched for genes associated with immunological processes (e.g., adaptive immune response, cytokine and interferon signaling, and T-cell receptor signaling) (Figure 4). This suggests that CNAs within the tumors that have high CNA burden can transcriptionally activate multiple processes that are linked to poor immune response by simultaneously influencing cell cycle and immune-evasive mechanisms to support tumor growth. We then predicted the phenotypic effects of altered expression levels of the genes in the “immune cluster” from our cofunctionality network in combination with a gene set collection obtained from the Mammalian Phenotype Ontology database, as previously published [34]. Interestingly, the altered expression levels of these genes were predicted to result in decreased abundances of T cells, B cells, CD8+ T cells, and interferon gamma secretion but an increased abundance of neutrophils (Figure 4).

### 3.6. Copy Number Alterations Included in Biomarker-Driven Clinical Trial Designs from the AACR GENIE Database

Given that the 78 CNAs found to be characteristic of each subgroup and their associated pathways could inform better strategies to match patients to personalized therapy, we sought to investigate their incorporation in biomarker-driven clinical trials. We reviewed data from the AACR Project GENIE Consortium [2], a source compiled from 148,268 patients (168,423 samples), to identify clinical trials that used these CNAs in the biomarker criteria of their prospective designs. For CNAs that were found to be significantly higher in the subgroup, “LowTMB/HighCNA”, we identified several amplifications and losses mainly related to cell cycle (e.g., *CDK4/6* amplifications and *CDKN2A/B* losses) and receptor tyrosine kinase pathway (e.g., *EGFR* amplification and *NF1/2* losses) to be included as biomarkers in clinical trial designs (Table 2). Most of the trials incorporating alterations in cell cycle pathway included treatments with CDK4/6 inhibitors, while those related to receptor tyrosine kinase were targeted with various kinase inhibitors (Table 2). The full list of these molecularly driven clinical trials and their details regarding the tumor cohorts, phases, and therapies evaluated are described in Supplementary Table S7. Among the CNAs that characterized the subgroup “HighTMB/HighCNA”, we identified four that were included as biomarkers in clinical trial designs, and these were related to PI3K/AKT1/mTOR pathway and chromatin remodeling/DNA methylation, which were mainly targeted using PI3K/mTOR and EZH2 inhibitors, respectively (Table 2 and Supplementary Table S7).

## 4. Discussion

In this paper, we present the results of a correlative analysis testing our primary hypothesis that an increased CNA burden predicts those patients who do not achieve a survival benefit on immunotherapy in the advanced setting through using a large, publicly available dataset of 1661 tumors profiled by the FDA-approved targeted assay of MSK-IMPACT. We further demonstrated our secondary hypothesis that the combinatorial biomarkers of TMB and CNA burden better stratify patients into subsets with differential response to ICB. These subsets were found to display distinct clinical outcomes, unique CNA profiles, enriched pathways, and predicted phenotypic effects related to immune signaling and CD8+ T-cell infiltration in the TME.

While our main observations that CNA burden is a predictor of resistance to immunotherapy is consistent with recent findings from studies investigating melanoma, NSCLC, and gastrointestinal tumors [25,27,28,29,30], these studies were mostly based on whole-exome sequencing methods not routinely utilized in clinical care as is the MSK-IMPACT. Thus, our analysis proposing the use of a current and already FDA-approved platform to determine CNA burden in concert with TMB has the advantage of providing critical information with a direct translation to refine current guidelines that utilize the same platform to assign patients to immunotherapy based on TMB only. These findings further shed the light on TMB as an imperfect predictive biomarker for immunotherapy response [9,19], as only a fraction of somatic mutations in DNA ultimately give rise to sufficient quantities of antigens containing neopeptides that elicit an effective antitumor immune response [19,37]. Moreover, our findings highlight the importance of assessing TMB while taking into account the overall genetic alterations of copy number gains and losses that could influence immune response through activating several immune evasive mechanisms in the TME. In this context, several individual CNAs that were found to be significantly higher in the subgroups characterized with “high CNA burden” in our analysis have been reported to be linked to an immunosuppressive TME that may confer immune resistance [38]. These observations were further illustrated in our cofunctionality network analysis of these individual alterations displaying a unique “immune cluster” predicted to result in decreased abundance of CD8+ T cells and interferon gamma signaling.

Our findings that the subgroup of “LowTMB/HighCNA”, which displayed the worst survival, had characteristic CNAs related to diverse pathways (e.g., cell cycle, receptor tyrosine kinase, DNA repair, TGFβ signaling, MAPK signaling, and MYC pathway) suggest that these tumors can simultaneously activate multiple oncogenic signaling processes while still lacking inflammation in the TME. These oncogenic processes have been shown to foster immunotherapy resistance through influencing several immune subsets in the TME, including the depletion of CD8+ T cells, the induction of regulatory T cells and myeloid-derived suppressor cells (MDSCs), the deregulation of interferon gamma signaling, and the upregulation of PD-L1 expression [23,39]. For instance, amplified MYC signaling can promote tumor proliferation, angiogenesis, and the creation of an immunosuppressive stroma that induces T-cell exclusion and dampens the capacity of antigen presentation [23,38]. Targeting MYC-dependent immune evasion has been proposed as a potential strategy to overcoming immunotherapy resistance to improve patients’ outcomes [40]. The cooperation of MYC signaling with other oncogenic processes such as RAS signaling further elicits immune suppression [41], which in turn drives immunotherapy resistance via multiple mechanisms, such as stabilizing mRNA expression [42] and activating the KRAS–IRF2 axis [43]. Altogether, these tumors may be classified as immunologically ignorant or excluded and may benefit from treatments targeting the immune stromal barrier elicited by oncogenic signaling along with immune augmentation strategies [44].

The observation that the “HighTMB/HighCNA” subgroup was more characterized with alterations related to epigenetic modifications (e.g., *EZH2* and *SUZ12*) and metabolic reprogramming when compared to the subgroup of “LowTMB/HighCNA” suggests that while these tumors exhibit high TMB and are more likely to form neoantigens, their antigen presentation ability may be hindered by epigenetic modifications known to downregulate HLA gene expression and subsequently induce several immune-resistance mechanisms via major histocompatibility complex (MHC) loss and T-cell exhaustion [23,45]. Alterations found to be characteristic of this subgroup affect components of the polycomb repressive complex 2 (PRC2) involved in chromatin silencing (e.g., *EZH2* and *SUZ12*) and could repress the transcription of MHC-I antigen-processing genes, rendering these tumors sensitive to strategies, such as use of EZH2 inhibitors, to restore antitumor immunity and elicit a response to ICB [46,47]. Furthermore, characteristic alterations in genes related to metabolic reprogramming such as succinate dehydrogenases (e.g., *SDHA*, *SDHC*) involved in the tricarboxylic acid cycle can lead to hypoxic induction of aerobic glycolysis that induces the expansion of immunosuppressive cells in the TME (e.g., regulatory T cells and MDSCs) and M2 macrophage polarization [10,48], suggesting that new combinations of metabolic reprogramming agents with ICB may promote immune activation and mitigate therapeutic resistance [23].

While significantly fewer alterations that are linked to an immunosuppressive TME were seen in the “LowTMB/LowCNA” subgroup, these tumors benefitted less from ICB when compared to those of the “HighTMB/LowCNA” subgroup, likely due to the low burden of somatic mutations that these tumors possess, resulting in a low neoantigenic load and thus making them immunologically cold and invisible to T cells to elicit tumor killing [23]. These tumors may have a pre-existing phenotype with a selection of tumor clones characterized by a paucity of immunogenic neoantigens [23], suggesting that these tumors may be better targeted with immune augmentation strategies such as adoptive T cells and cancer vaccines or potential natural-killer-based therapies that do not require neoantigen presentation to improve immunotherapy efficacy [23].

Our findings may be of a clinical value in guiding therapeutic choices in advanced cancers, as we provide evidence to hypothesize that “HighTMB/LowCNA” status better defines the tumors that respond to ICB harboring a greater mutational load and an increased likelihood of recognition by neoantigen-reactive T cells. This “immune hot” subgroup accounted for nearly 14% of the cases included in our analysis of the translational MSK-IMPACT cohort, which is close to the overall (<15%) average fraction of patients with various advanced cancers reported to benefit from immunotherapy [49]. Indeed, resistance to immunotherapy is still seen over an extended period of follow-up for most of patients with advanced cancers, and therefore improving their survival rates prevails as a high priority for the immuno-oncology community [18]. The identification of distinct subgroups and their unique 78 individual CNAs in this study highlights that incorporating CNA burden along with TMB assessment may better inform treatment decisions for immunotherapy or the prioritization of alternative strategies in clinical practice. In this context, several pancancer initiatives completed thus far apart from the MSK-IMPACT, such as NCI-MATCH [50], K-MASTER [51], and BC Cancer Personalized OncoGenomics [52], as well as other ongoing clinical trial programs, such as TAPUR (NCT02693535) and CAPTUR (NCT03297606), have been characterizing the complex genomic makeup of advanced tumors to identify druggable changes to match patients for improved personalized treatments in the clinical setting. While the majority of the molecularly driven biomarker-defined subsets found to be responsive to specific targets in these studies are commonly mutation-based, many potential amplification/loss targets were identified as well. Indeed, our review of the large, publicly accessible registry of the AACR-GENIE Project Consortium, including genomic data of 168,423 samples [2], revealed several of the key individual CNAs identified in our study that can be incorporated in the prospective design of clinical trials, which may have the potential to target specific histologies.

The association between high CNA burden and immunotherapy benefit observed in glioma that was distinct from other cancer types might be explained by the previous exposure of these tumors to the alkylating agent temozolomide, which has been shown to benefit gliomas harboring several CNAs [53]. Furthermore, the original analysis of the same MSK-IMPACT dataset showed that high TMB was associated with poor survival after immunotherapy in gliomas, suggesting that the previous exposure of these tumors to temozolomide can promote less immunogenic subclonal mutations and confer immunotherapy resistance [17].

Our study has several limitations. First, while we used the MSK-IMPACT cohort, which is a large, publicly available dataset of patients treated with ICB, the small number of cases classified with high TMB included in tumors other than melanoma, NSCLC, and bladder cancer might have decreased the power to observe significant findings for the predictive capacity of the “TMB/CNA" subgroups for immunotherapy response within these tumors. Second, unlike the prespecified FDA-approved cut-point of 10 mut/mb followed in this study to define high TMB tumors, we used the median cut-point within each tumor type to define high CNA burden since validated cut-points for CNA burden are currently lacking. Nonetheless, we followed a similar approach to the original MSK-IMPACT study, which initially demonstrated the capacity of TMB to predict immunotherapy benefit using the median cut-point of TMB burden prior to the subsequent validation and development of the universal tissue-agnostic 10 mut/mb. In a similar fashion, our study provides a proof-of-concept required to train and validate a tissue-agnostic universal cut-point for CNA burden that is more likely to be translated into clinic. While previous attempts using MSK-IMPACT data for immunotherapy response have proposed methods, such as elbow-point, that are based on all cases or the lower tertile in the NSCLC cohort to determine the cut-off used for calling CNAs [54,55], analytical variability issues related to the assessment of CNA burden still need to be optimized and standardized to reach a consensus on the best modality for defining tumors with high CNA burden. Lastly, while we identified 78 individual CNAs to be characteristic of four clinically distinct subgroups with a potential to be included in biomarker-driven clinical trial designs, other CNAs might also be of interest and should be further investigated in additional large cohorts of patients treated with ICB.

## 5. Conclusions

In this study we demonstrated the predictive capacity of CNA burden to be a biomarker for immunotherapy resistance on a large dataset of 1661 cases spanning different advanced cancer types and using an FDA-approved test available in clinical practice. We further showed that the combination of TMB and CNA burden better stratifies patients into subsets with differential response to ICB with unique CNA profiles, enriched pathways, and predicted transcriptional and phenotypic effects related to immune signaling and CD8+ T-cell abundance in the tumor microenvironment. These findings may better guide patient selection for immunotherapy or alternative/matched strategies in clinical practice.

## Figures and Tables

**Table 1:** Characteristics of the MSK-IMPACT translational study cohort.

Variable ^1^	*n* (%)
All tumors	1661
Cancer type	
Melanoma	320 (19.27%)
Renal cell carcinoma	151 (9.09%)
Bladder cancer	215 (12.94%)
Breast cancer	44 (2.65%)
HR^+^/HER2^−^	17
HR^+^/HER2^+^	4
HR^−^/HER2^+^	3
Triple negative	6
Non-small-cell lung cancer	350 (21.07%)
Colorectal cancer	110 (6.62%)
Head and neck cancer	139 (8.37%)
Esophagogastric cancer	126 (7.59%)
Glioma	117 (7.04%)
Cancer of unknown primary	88 (5.30%)
Skin cancer, nonmelanoma	1 (0.06%)
Drug class	
Anti-PD-1/PD-L1	1307 (78.69%)
Anti-CTLA-4	99 (5.96%)
Combination	255 (15.35%)
Sample type	
Primary	731 (44%)
Metastasis	930 (56%)
Age group (years)	
<30	50 (3.01%)
31–50	283 (17.05%)
50–60	416 (25.04%)
61–70	499 (30.04%)
>71	413 (24.86%)
Smoking history	
Previous/current smoker	443 (26.67%)
Never	462 (27.81%)
Unknown	756 (45.52%)
Sex	
Male	1034 (62.25%)
Female	627 (37.75%)
TMB category	
High (≥10 mut/mb)	499 (30.04%)
Low (<10 mut/mb)	1162 (69.96%)

^1^ Abbreviations: HR—hormone receptor; HER2—human epidermal growth factor receptor 2; PD-1—programmed cell death protein 1; PD-L1—programmed cell death protein ligand 1; CTLA-4–cytotoxic T-lymphocyte associated protein 4; TMB—tumor mutational burden.

**Table 2:** Gene alterations characteristic of the “High-CNA” burden subgroups included in the molecularly driven biomarker clinical trial designs ^1^.

Subgroup	Pathway	Alterations Included in Molecularly Driven Biomarker Clinical Trial Designs	Therapies Tested
LowTMB/ HighCNA	Cell cycle pathway		
		*CDKN2A* loss	Palbociclib, abemaciclib, ribociclib (CDK4/6 inhibitor) Ilorasertib (Aurora and VEGFR kinase inhibitor)
		*CDKN2B* loss	Abemaciclib, ribociclib (CDK4/6 inhibitor)
		*CDK4* amplification	PF-07220060 (CDK4 inhibitor)
		*CDK6* amplification	Palbociclib, abemaciclib, ribociclib (CDK4/6 inhibitor)
		*RB1* loss	Abemaciclib (CDK4/6 inhibitor) Prexasertib, SRA737 (CHK1 inhibitor) Apalutamide (AR antagonist) with cetrelimab (PD-1 inhibitor) Ceterlimab (PD-1 inhibitor) with carboplatin, cabazitaxel, and niraparib Valemetostat (EZH2 inhibitor) and ipilimumab Osimertinib (tyrosine kinase inhibitor) Olaparib (PARP inhibitor)
	Receptor tyrosine kinase pathway		
		*ALK* amplification	Belantamab mafodotin (B-cell maturation antigen inhibitor) AUY922 (HSP90 inhibitor) Ceritinib, ensartinib (ALK kinase inhibitor) Crizotinib, entrectinib (multitargeted tyrosine kinase inhibitor) TSR-011 (ALK and tropomyosin kinase inhibitor)
		*EGFR* amplification	BCA101 (TGF-β and EGFR inhibitor) Osimertinib, GC1118, nimotuzumab, Sym004, gefitinib (EGFR tyrosine kinase inhibitor) Sunitinib (multitargeted tyrosine kinase inhibitor) Afatinib (ErbB family blocker) Capivasertib (pan AKT kinase inhibitor) Depatuxizumab mafodotin (EGFR antibody–drug conjugate) Paziotinib (pan HER2 inhibitor) Lapatinib (EGFR and HER2 inhibitor) MSC2363318A (AKT dual inhibitor) PF-00299804 (pan HER inhibitor)
		*ERBB3* amplification	Zotatifin (HER3 blocker) Pertuzumab (HER2-HER3 inhibitor)
		*FGFR4* amplification	Rogaratinib (FGFR4 inhibitor) Infigratinib, Erdafitinib, pemigatinib (FGFR kinase inhibitor) Debio-1347, E7090, derazantinib (FGFR 1–3 inhibitor)
		*FLT1* amplification	Regorafenib (multikinase inhibitor) Nintedanib (angiokinase inhibitor)
		*FLT3* amplification	Regorafenib (multikinase inhibitor) Nintedanib (angiokinase inhibitor) Ponatinib (multikinase inhibitor)
		*KDR* amplification	Regorafenib (multikinase inhibitor) Nintedanib (angiokinase inhibitor)
		*NF1* loss	Selumetinib, trametinib, cobimetinib, mirdametinib, binimetinib (MEK inhibitor) RMC-4630 (SPH2 inhibitor) Temsirolimus, everolimus (mTOR inhibitor) LY3214996 (ERK inhibitor)
		*NF2* loss	Selumetinib, mirdametinib (MEK inhibitor) Temsirolimus, everolimus, AZD2014 (mTOR inhibitor) Axitinib (VEGFR tyrosine kinase inhibitor) Lapatinib (EGFR and HER2 inhibitor)
		*PDGFRA* amplification	Crenolanib (PDGFR inhibitor) Ripretinib (PDGFRA and kit inhibitor) Sitravatinib, ponatinib, sunitinib, dasatinib, regorafinib (multikinase inhibitor) Nilotinib (tyrosine kinase inhibitor) Nab-Rapamycin (mTOR inhibitor) Capivasertib (pan AKT kinase inhibitor) Samotolisib (PI3K and mTOR inhibitor)
	mTOR pathway		
		*FLCN* loss	Temsirolimus (mTOR inhibitor)
	p53 pathway		
		*MDM2* amplification	Idasanutlin, ALRN-6924, BI 907828, AMG 232 (MDM2 inhibitor)
	MYC pathway		
		*MYC* amplification	Fadraciclib (CDK2/9 inhibitor) KB-0742 (CDK9 inhibitor) ORIN1001 (IRE1 RNase inhibitor suppressing MYC high tumors) BMS-986158 (BET inhibitor) Berzosertib (ATR inhibitor) MIK665 (MCL1 inhibitor) Prexasertib, SRA737 (CHK1 inhibitor) Telaglenastat (glutaminase inhibitor) RMC-5552 (mTORC1 inhibitor)
HighTMB/ HighCNA	Chromatin remodeling/ DNA methylation		
		*EZH2* amplification	Tazemetostat (EZH2 inhibitor)
	Apoptosis pathway		
		*MCL1* amplification	Fadraciclib (CDK2/9 inhibitor)
	PI3K/AKT1/ mTOR pathway		
		*PTEN* loss	RMC-5552 (mTORC1 inhibitor) Copanlisib, alpelisib, paxalisib, capivasertib AZD8186, GSK2636771 (PI3K inhibitor) Ipatasertib, TAS-117 (AKT1/2/3 kinase inhibitor) TAS0612 (AKT/RSK/S6K inhibitor) Gedatolisib (PI3K and mTOR inhibitor) Temsirolimus, everolimus (mTOR inhibitor) Sapanisertib (mTORC1 and mTORC2 inhibitor) Sirolimus (mTOR inhibitor) Vistusertib (mTOR 1,2 inhibitor) PQR309 (PI3K/mTOR inhibitor) MSC2363318A (AKT1,3 and p70S6K inhibitor) Samotolisib (PI3K and mTOR inhibitor) Sunitinib (multitargeted tyrosine kinase inhibitor) Buparilisib (pan PI3K inhibitor) Niraparib, olaparib (PARP inhibitor)
		*RICTOR* amplification	RMC-5552 (mTORC1 inhibitor) Onatasertib, vistusertib (mTOR 1,2 inhibitor) Paxalisib, godatolisib (PI3K/mTOR inhibitor) Everolimus (mTOR inhibitor) Ipatasertib, capivasertib, TAS-117 (AKT1/2/3 kinase inhibitor) Samotolisib (PI3K and mTOR inhibitor) Nab-Rapamycin (mTOR inhibitor) GSK2636771 (PI3K inhibitor)

^1^ Full details on the molecularly driven biomarker clinical trials retrieved from the AACR Project GENIE Consortium database; their cohorts and designs can be found in Supplementary Table S7. TMB—tumor mutational burden; CNA—copy number alteration.

## Data Availability

This study involved the collection and analysis of data from publicly available datasets as detailed in the Data Availability Section within the Methods. The MSK-IMPACT data can be accessed via the cBioPortal for Cancer Genomics repository (https://www.cbioportal.org/, accessed on 15 June 2023—unique identifier: “TMB and Immunotherapy (MSK, Nature Genetics 2019)”); Supplementary Data were obtained from Samstein et al. in Nat. Genet. 2019 [17] and Zehir et al. Nat. Med. [16]. Data investigating CNAs included in biomarker-driven clinical trials in the AACR Project GENIE Consortium [2] can be accessed via https://www.mycancergenome.org (accessed on 13 October 2023). No custom code was generated to collect data in this study. Any queries for R codes used in the analyses of this study should be directed to the corresponding author.

## Supplementary Materials

The following supporting information can be downloaded at https://www.mdpi.com/article/10.3390/cancers16040732/s1: Figure S1: Kaplan–Meier curves showing overall survival for cancer patients stratified according to CNA burden categories in all cases and in (C) the melanoma cohort, (D) bladder cancer cohort, (E_ NSCLC cohort, and (F) colorectal cancer cohort; Figure S2: Alluvial plot showing the distribution of the four subgroups stratified according to the combinatorial expression of TMB and CNA burden across the different cancer types; Figure S3: Stack plot showing the frequency of the copy number alterations in the 78 genes found to be characteristic of the four “TMB/CNA" subgroups according to cancer type; Figure S4: Circulatory heatmaps showing the association between individual CNAs and the immune cell abundance of CD8+ T cells evaluated in The Cancer Genome Atlas (TCGA) cohorts of breast cancer and esophagogastric cancer; Table S1: Multivariate analysis for the continuous increase in CNA burden as determined by the fraction of genome altered scores, with their association being tested with overall survival after immune checkpoint blockade across different tumor types; Table S2: Distribution of the four subgroups stratified according to the combinatorial expression of TMB and CNA burden across the different cancer types; Table S3: Characteristics of the four “TMB/CNA” subgroups identified in the MSK-IMPACT translational study cohort; Table S4: Genes with individual copy number alterations characteristic of at least one “TMB/CNA” subgroup vs. others; Table S5: A list of 78 genes whose alterations significantly distinguish at least one “TMB/CNA” subgroup vs. others and their associated pathways; Table S6: Association between the 78 gene copy number alterations that characterize the “TMB/CNA” subgroups with CD8+ T-cell abundance; Table S7: Gene alterations that characterize the subgroups with high CNA burden included in biomarker-driven clinical trial designs (retrieved from the AACR GENIE Project Consortium database).
