# Supplementary material for: Tumor Copy Number Alteration Burden as a Predictor for Resistance to Immune Checkpoint Blockade across Different Cancer Types
Source: Cancers (Basel). 2024 Feb 9;16(4):732. doi: 10.3390/cancers16040732 (PMC10886982; doi:10.3390/cancers16040732)

Supplementary Figure S1

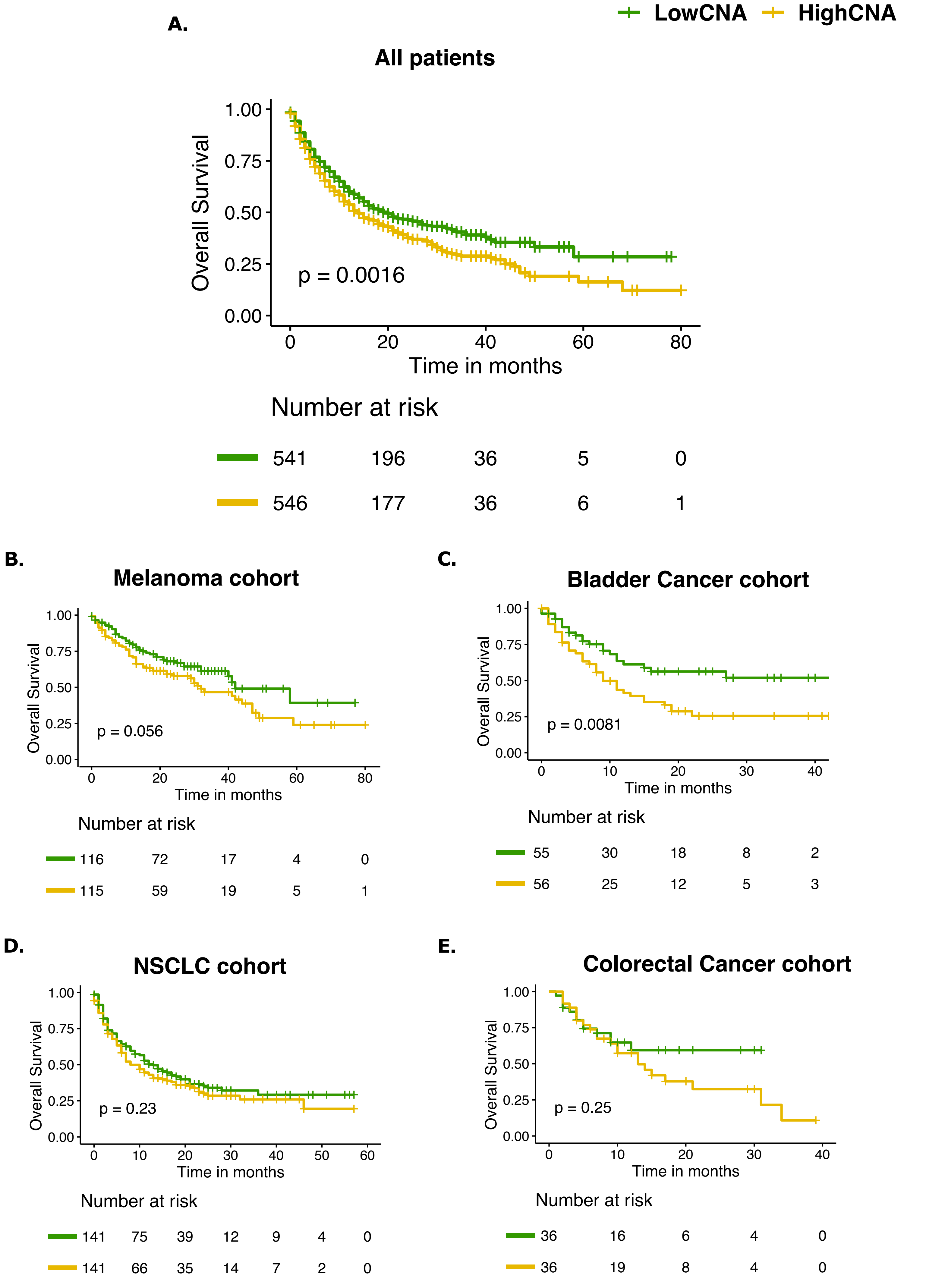

Supplement: Supplementary file 1 [file cancers-16-00732-s001.zip › Supplementary_Figures/Supplementary_Figure_S1.pdf]
