# Supplementary material for: Tumor Copy Number Alteration Burden as a Predictor for Resistance to Immune Checkpoint Blockade across Different Cancer Types
Source: Cancers (Basel). 2024 Feb 9;16(4):732. doi: 10.3390/cancers16040732 (PMC10886982; doi:10.3390/cancers16040732)
Supplement: Supplementary file 1 [file cancers-16-00732-s001.zip › Supplementary_Tables/Supplementary Table S1.pdf]

Supplementary Table S1. Multivariate analysis for the continuous increase in CNA burden, as determined by fraction of genome altered scores, testing their association with overall survival after immune checkpoint blockade across different tumor types.

Multivariate analysis for overall survival after immune checkpoint inhibitor treatment across different tumor types*					
Univariate analysis			Multivariate analysis		
**Cancer Type**	**HR (95%CI)**	***P*-value**	**Cancer Type**	**HR (95%CI)**	***P*-value**
All samples	1.37 (0.92-2.1)	0.13	All samples	1.52 (1.01-2.30)	0.04
Melanoma	0.80 (0.87-5.69)	0.10	Melanoma	2.59 (0.99-6.76)	0.05
Renal Cell Carcinoma	2.94 (0.71-12.25)	0.14	Renal Cell Carcinoma	2.42 (0.56-10.41)	0.23
Bladder Cancer	3.71 (0.27-0.96)	0.06	Bladder Cancer	5.42 (1.29-22.80)	0.02
Breast Cancer	0.50 (0.09-2.80)	0.43	Breast Cancer	0.98 (0.11-8.96)	0.98
Non−Small Cell Lung Cancer	1.06 (0.51-2.21)	0.88	Non−Small Cell Lung Cancer	0.90 (0.41-1.97)	0.80
Colorectal Cancer	5.48 (0.71-42.24)	0.10	Colorectal Cancer	3.99 (0.42-38.05)	0.23
Head and Neck Cancer	3.74 (0.64-21.74)	0.14	Head and Neck Cancer	5.63 (0.85-37.38)	0.07
Esophagogastric Cancer	0.72 (0.13-3.99)	0.70	Esophagogastric Cancer	0.56 (0.09-3.68)	0.55
Glioma	0.15 (0.02-1.03)	0.05	Glioma	0.14 (0.02-1.008)	0.05
Cancer of Unknown Primary	0.97 (0.06-16.44)	0.98	Cancer of Unknown Primary	0.49 (0.02-14.06)	0.7

*Skin Cancer, Non−Melanoma had only 1 case and was not analyzed. Depiction of the multivariate results is found in Figure 2A.
