# Supplementary material for: Tumor Copy Number Alteration Burden as a Predictor for Resistance to Immune Checkpoint Blockade across Different Cancer Types
Source: Cancers (Basel). 2024 Feb 9;16(4):732. doi: 10.3390/cancers16040732 (PMC10886982; doi:10.3390/cancers16040732)
Supplement: Supplementary file 1 [file cancers-16-00732-s001.zip › Supplementary_Tables/Supplementary Table S2.pdf]

Supplementary Table S2. Distribution of the four subgroups stratified according to the combinatorial expression of TMB and CNA burden across the different cancer types.

Cancer Type	Frequency	TMB/CNA subgroup
Melanoma	63	LowTMB/LowCNA
Melanoma	54	LowTMB/HighCNA
Melanoma	53	HighTMB/LowCNA
Melanoma	61	HighTMB/HighCNA
Bladder Cancer	33	LowTMB/LowCNA
Bladder Cancer	28	LowTMB/HighCNA
Bladder Cancer	22	HighTMB/LowCNA
Bladder Cancer	28	HighTMB/HighCNA
Breast Cancer	16	LowTMB/LowCNA
Breast Cancer	17	LowTMB/HighCNA
Breast Cancer	1	HighTMB/LowCNA
Breast Cancer	1	HighTMB/HighCNA
Colorectal Cancer	17	LowTMB/LowCNA
Colorectal Cancer	26	LowTMB/HighCNA
Colorectal Cancer	19	HighTMB/LowCNA
Colorectal Cancer	10	HighTMB/HighCNA
Esophagogastric Cancer	22	LowTMB/LowCNA
Esophagogastric Cancer	27	LowTMB/HighCNA
Esophagogastric Cancer	8	HighTMB/LowCNA
Esophagogastric Cancer	4	HighTMB/HighCNA
Glioma	42	LowTMB/LowCNA
Glioma	40	LowTMB/HighCNA
Glioma	2	HighTMB/LowCNA
Glioma	5	HighTMB/HighCNA
Head and Neck Cancer	31	LowTMB/LowCNA
Head and Neck Cancer	29	LowTMB/HighCNA
Head and Neck Cancer	6	HighTMB/LowCNA
Head and Neck Cancer	9	HighTMB/HighCNA
Non-Small Cell Lung Cancer	100	LowTMB/LowCNA
Non-Small Cell Lung Cancer	83	LowTMB/HighCNA
Non-Small Cell Lung Cancer	41	HighTMB/LowCNA
Non-Small Cell Lung Cancer	58	HighTMB/HighCNA
Renal Cell Carcinoma	62	LowTMB/LowCNA
Renal Cell Carcinoma	65	LowTMB/HighCNA
Renal Cell Carcinoma	3	HighTMB/LowCNA
Renal Cell Carcinoma	1	HighTMB/HighCNA
