# Supplementary material for: Tumor Copy Number Alteration Burden as a Predictor for Resistance to Immune Checkpoint Blockade across Different Cancer Types
Source: Cancers (Basel). 2024 Feb 9;16(4):732. doi: 10.3390/cancers16040732 (PMC10886982; doi:10.3390/cancers16040732)
Supplement: Supplementary file 1 [file cancers-16-00732-s001.zip › Supplementary_Tables/Supplementary Table S3.pdf]

Supplementary Table S3. Characteristics of the four 'TMB/CNA' subgroups identified in the MSK-IMPACT translational study cohort.

	**LowTMB/ LowCNA (n=386)**	**LowTMB/ HighCNA (n=369)**	**HighTMB/ LowCNA (n=155)**	**HighTMB/ HighCNA (n=177)**	***P*-value**
**Sample type**					
Primary	193 (50%)	186 (50.4%)	63 (40.6%)	59 (33.3%)	<0.001
Metastasis	193 (50%)	183 (49.6%)	92 (59.4%)	118 (66.7%)	
**Drug class**					
Anti PD-1/PD-L1	289 (75%)	288 (78%)	111 (71.6%)	129 (73%)	0.36
Anti CTLA-4	28 (7.2%)	24 (6.5%)	19 (12.3%)	16 (9%)	
Combination	69 (17.8%)	57 (15.5%)	25 (16.1%)	31 (17.5%)	
**Sex**					
Male	228 (59%)	227 (61.5%)	97 (62.6%)	111 (62.7%)	0.78
Female	158 (41%)	142 (38.4%)	58 (37.4%)	66 (37.3%)	
**Age group**					
≤30	18 (4.7%)	10 (2.7%)	2 (1.3%)	3 (1.7%)	<0.01
31-50	83 (21.5%)	74 (20%)	16 (10.3%)	24 (13.6%)	
50-60	100 (25.9%)	98 (26.6%)	38 (24.5%)	42 (23.7%)	
61-70	103 (26.7%)	111 (30.1%)	46 (29.7%)	63 (35.6%)	
≥71	82 (21.2%)	76 (21.6%)	53 (34.2%)	45 (25.4%)	
**Smoking history**					
Previous/Current smoker	153 (39.6%)	147 (39.8%)	60 (38.7%)	75 (42.4%)	0.82
Never	162 (42%)	156 (42.3%)	71 (45.8%)	66 (37.3%)	
Unknown	71 (18.4%)	66 (17.9%)	24 (15.5%)	36 (20.3%)	

Abbreviations: TMB, tumor mutational burden; CNA, copy number alterations burden.
