# Supplementary figures and images for: Tumor Copy Number Alteration Burden as a Predictor for Resistance to Immune Checkpoint Blockade across Different Cancer Types
Source: Cancers (Basel). 2024 Feb 9;16(4):732. doi: 10.3390/cancers16040732 (PMC10886982; doi:10.3390/cancers16040732)

Supplementary Figure S2

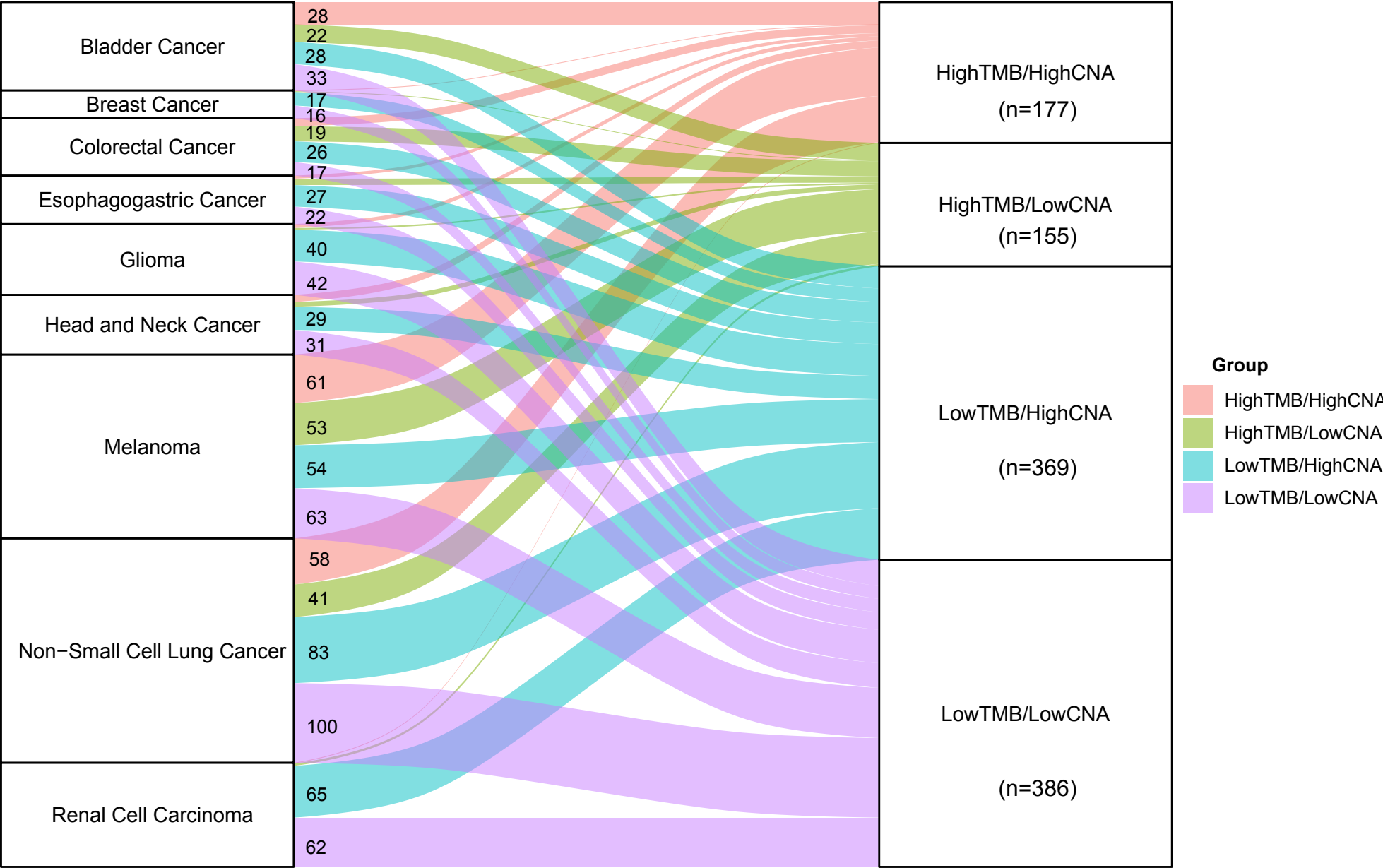

Supplement: Supplementary file 1 [file cancers-16-00732-s001.zip › Supplementary_Figures/Supplementary_Figure_S2.pdf]

### Supplementary Figure S3

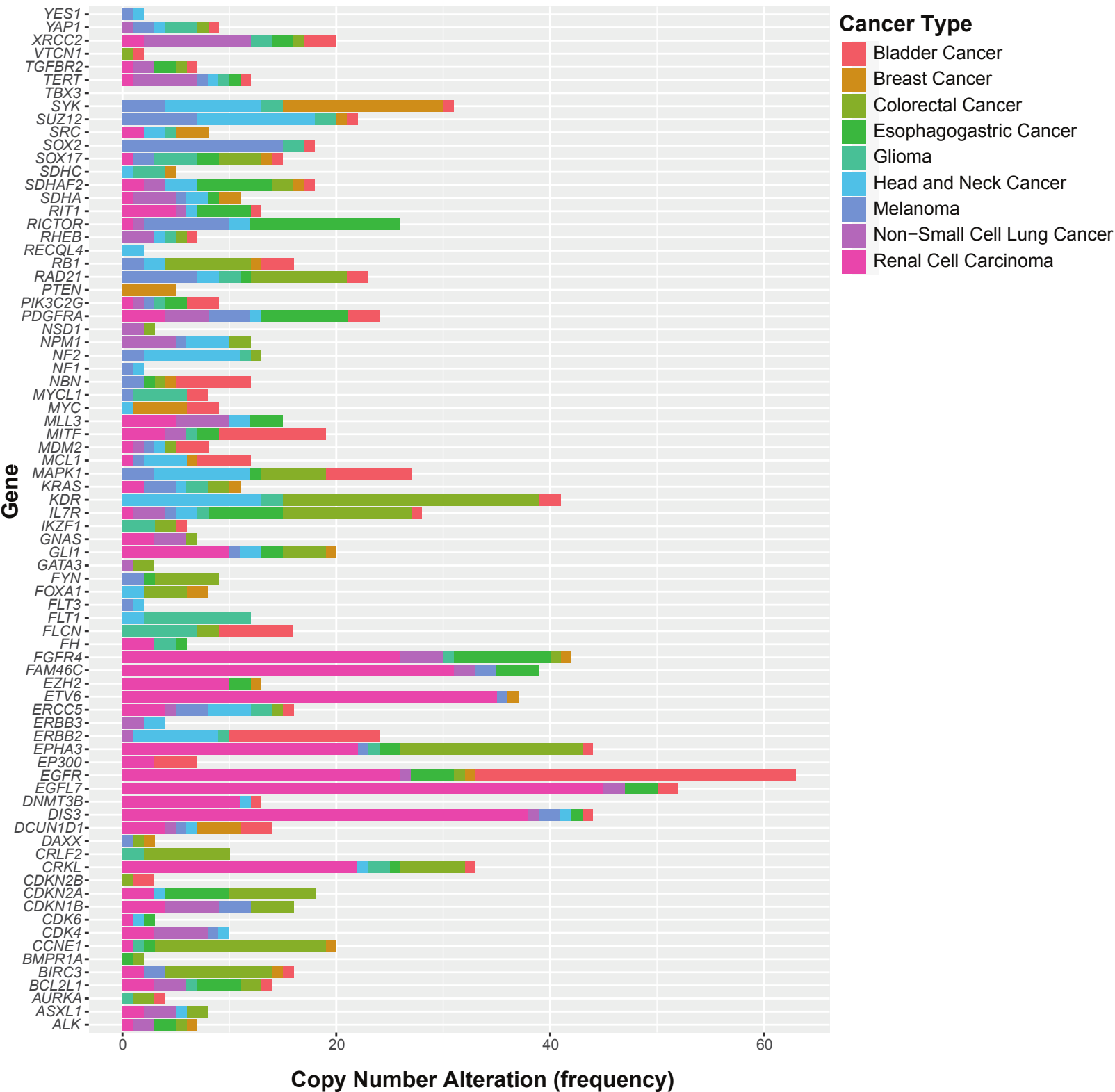

Supplement: Supplementary file 1 [file cancers-16-00732-s001.zip › Supplementary_Figures/Supplementary_Figure_S3.pdf]

Supplementary Figure S4

A.

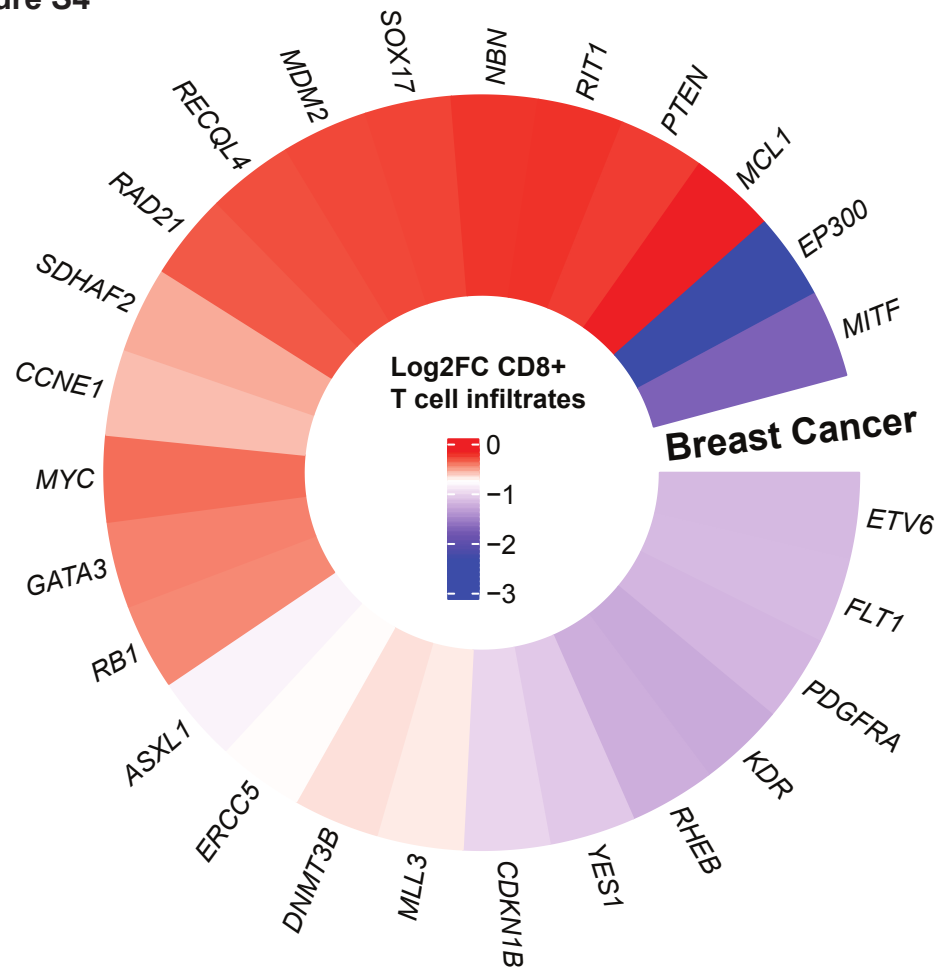

B.

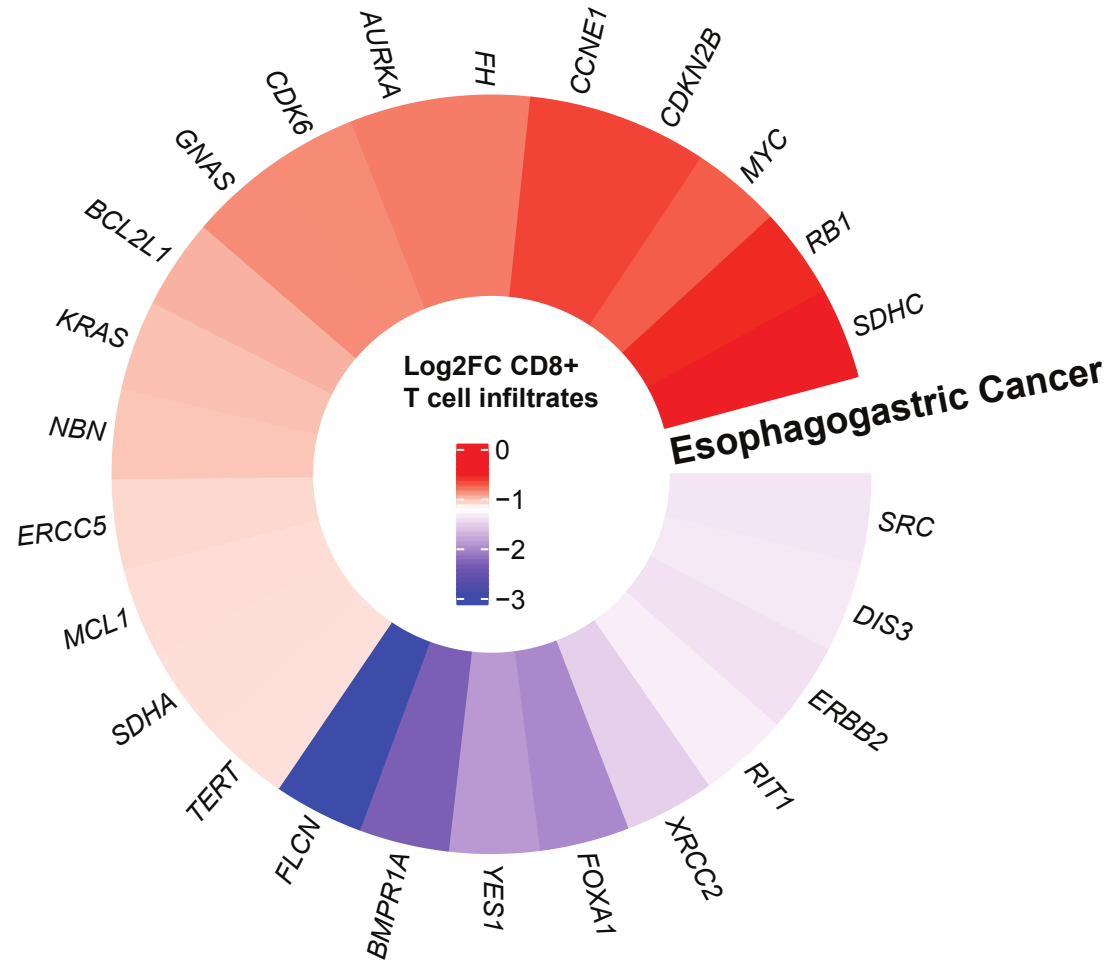

Supplement: Supplementary file 1 [file cancers-16-00732-s001.zip › Supplementary_Figures/Supplementary_Figure_S4.pdf]
